# Fine Dissection of Human Mitochondrial DNA Haplogroup HV Lineages Reveals Paleolithic Signatures from European Glacial Refugia

**DOI:** 10.1371/journal.pone.0144391

**Published:** 2015-12-07

**Authors:** Sara De Fanti, Chiara Barbieri, Stefania Sarno, Federica Sevini, Dario Vianello, Erika Tamm, Ene Metspalu, Mannis van Oven, Alexander Hübner, Marco Sazzini, Claudio Franceschi, Davide Pettener, Donata Luiselli

**Affiliations:** 1 Department of Biological, Geological and Environmental Sciences, University of Bologna, Bologna, Italy; 2 Department of Experimental, Diagnostic and Specialty Medicine, University of Bologna, Bologna, Italy; 3 C.I.G. Interdepartmental Centre L. Galvani for Integrated Studies on Bioinformatics, Biophysics and Biocomplexity, University of Bologna, Bologna, Italy; 4 Estonian Biocentre, Evolutionary Biology group, Tartu, Estonia; 5 Department of Evolutionary Biology, University of Tartu, Tartu, Estonia; 6 Department of Forensic Molecular Biology, Erasmus MC - University Medical Center Rotterdam, Rotterdam, The Netherlands; 7 Department of Evolutionary Genetics, Max Planck Institute for Evolutionary Anthropology, Leipzig, Germany; 8 IRCCS, Institute of Neurological Sciences of Bologna, Ospedale Bellaria, Bologna, Italy; 9 CNR, Institute of Organic Synthesis and Photoreactivity (ISOF), Bologna, Italy; University College Dublin, IRELAND

## Abstract

Genetic signatures from the Paleolithic inhabitants of Eurasia can be traced from the early divergent mitochondrial DNA lineages still present in contemporary human populations. Previous studies already suggested a pre-Neolithic diffusion of mitochondrial haplogroup HV*(xH,V) lineages, a relatively rare class of mtDNA types that includes parallel branches mainly distributed across Europe and West Asia with a certain degree of structure. Up till now, variation within haplogroup HV was addressed mainly by analyzing sequence data from the mtDNA control region, except for specific sub-branches, such as HV4 or the widely distributed haplogroups H and V. In this study, we present a revised HV topology based on full mtDNA genome data, and we include a comprehensive dataset consisting of 316 complete mtDNA sequences including 60 new samples from the Italian peninsula, a previously underrepresented geographic area. We highlight points of instability in the particular topology of this haplogroup, reconstructed with BEAST-generated trees and networks. We also confirm a major lineage expansion that probably followed the Late Glacial Maximum and preceded Neolithic population movements. We finally observe that Italy harbors a reservoir of mtDNA diversity, with deep-rooting HV lineages often related to sequences present in the Caucasus and the Middle East. The resulting hypothesis of a glacial refugium in Southern Italy has implications for the understanding of late Paleolithic population movements and is discussed within the archaeological cultural shifts occurred over the entire continent.

## Introduction

In the past years, an increasing number of studies reported whole mitochondrial genome data from various human populations from all the continents, providing new insights into the shape of the matrilineal phylogeny and the distribution of its clades. These fine-grained studies helped to both understand demic migration flows and disentangle the phylogeographic distribution and the time of divergence of characteristic mitochondrial DNA (mtDNA) lineages [1–8]. Uniparental genetic markers (i.e. mtDNA and the non-recombinant portion of the Y chromosome) have been widely studied for reconstructing the prehistory of European and Mediterranean populations from living individuals [9–13], as well as from ancient DNA (aDNA) samples [13]. Accordingly, the available published data from these regions provide an extensive geographical coverage, even though at a shallow level of phylogenetic resolution. In fact, until a couple of years ago, population-level analyses of mtDNA focused almost exclusively on the hypervariable segment(s) (HVS) located within the D-loop or control region. Only recently, this kind of studies began to include full mtDNA genome data to explore the variability of specific lineages at a higher resolution and to get deeper insights into the matrilineal prehistory of a given population [5, 14–16].

A recent comprehensive survey of the Italian uniparental genetic landscape, comprising almost a thousand of individuals, revealed that mtDNA haplogroup HV is the lineage in Italy with the oldest coalescence, and highlighted a divergent structure between the Northern and the Southern regions of the peninsula [17]. This early signal can be paralleled with noticeable findings obtained by aDNA analysis on remains from a Mesolithic site in Sicily (Favignana, 14 kilo years ago, kya) [18]), which included a specimen assigned to haplogroup HV1. A Paleolithic site in Puglia (Paglicci, 28 kya) also reported data from an individual assigned to HV* (or pre-HV/R0) [19], but such finding could not be replicated in a more recent study [20]. While haplogroup HV was recognized as a crucial component of early human dispersal in Eurasia [9,21], patterns of its internal variability have been poorly investigated in previous works, which only focused on the major clades HV4 [4], V and H [21–27].

Haplogroup HV is a major mitochondrial clade within haplogroup R0 [9] characterized by the T14766C mutation and comprising at least 18 recognized basal subclades. Seventeen of these are designated with a consecutive numerical label, i.e. HV0-HV17 (the HV3 label is not used) (http://www.phylotree.org/, Build 16) [28]. Additionally, it includes haplogroup H as one of its direct subclades, while haplogroup V is a nested subhaplogroup within the HV0 clade. The basal structure of the HV phylogeny is partially characterized by mutations located within the hypervariable segments. Some of these branch-defining variants occur for example at nucleotide positions 72, 73, 152, 195, 16311, which are recognized as recurrent sites throughout the whole mtDNA phylogeny—the latter three for example, appears more than 80 times in the tree [29]—and could therefore obscure the topology of the reconstructed HV tree.

The HV clade as a whole, including its H subclade, encompasses Eurasian haplogroups that likely arose between Western and Central Asia [30]. Haplogroup HV*(xH,V) (i.e. the whole of HV excluding its subclades H and V) is not particularly common in Europe, with frequencies that range between 0% and 10%. In particular, high HV* occurrences are observed in Southern Europe (e.g., Italy and Spain) and exceptionally high frequencies are found in Iran (19–24%) [31], although somewhat lower values (9–14%) are reported for the same populations in Farjadian et al. [32]. An average frequency of 4.05% is observed in the Italian peninsula [17]. On the other hand, the major HV subclade, haplogroup H, experienced a vast diffusion during the late Neolithic time, becoming the most common haplogroup in Europe today [27,33]. Other lineages within haplogroup HV were likely spread in more ancient times, dating back to more than 15 kya and having independent diffusions within Europe of Early, Middle or Late Upper Paleolithic origins [9,21].

Several scholars associated the diffusion of Paleolithic characteristic lineages with the re-peopling of the European continent from refugia after the Late Glacial Maximum (LGM) ~27–16 kya [5,30,34,35]. These areas could have served as a reservoir of genetic variation for particular lineages, which would have subsequently expanded in Europe in synchrony with the amelioration of the climatic conditions, leaving detectable traces in the current lineage distribution. The major glacial refugia recognized from geological and ecological data (in particular from the genetics and distribution of key species of animals and plants) are the Franco-Cantabrian region, the Balkan-Caucasus, and Southern Italy [36–39].

Haplogroup HV4 was previously analyzed as a case study for this kind of phylogeographic reconstruction [4,40]. A strong signal of expansion detected in one of its sublineages, found at high frequency at the border between Northern Spain and France, was associated to the Franco Cantabrian post glacial re-population, showing genetic patterns in line with isolation followed by expansion. Gόmez-Carballa and colleagues propose that HV4 originated 14.2 kya in Eastern Europe and that its major sub-branch HV4a1a experienced a major expansion from the Basque region, where the majority of the examined sequences come from, during the Younger Dryas. Finally, a sparse presence of HV4 lineages in Southern Italy was explained by historical migrations that took place during the past centuries.

However, the lack of appropriate data representative of the entire Italian gene pool has long affected the description of these rare lineages, which are often selected within limited datasets and targeting specific subsets of variability. In this study, we analyze a non-biased (i.e. derived from a broad population sampling of the entire Italian territory) sample of 50 Italian individuals belonging to haplogroup HV within a total of 70 newly generated complete HV*(xH,V) mtDNA genomes, together with a dataset which reproduces the variability of the whole haplogroup HV at its current state of art, for a total of 316 mtDNA genomes. We thus aim at investigating patterns of diversity localized in time and space and at revising the fine grain variation within the whole macro haplogroup, which reconstructed phylogeny appears particularly unstable.

According to our results, we propose a more complete perspective on the diffusion and variability of haplogroup HV4, not exclusively centered on the Franco Cantabrian area. We also discover a clear signal of ancient structure in the pool of Italian HV lineages, which emerged from the identification of new autochthonous subhaplogroups, and which we contextualize with the transitional Mesolithic material cultures characteristic of these southern regions. Finally, the role of the Italian peninsula as a glacial refugium is investigated from an Anthropological Genetics perspective. Our interpretation of ancient structure suggests a pivotal role for Italy as a reservoir of diversity that survived until present time in a relic form.

## Material and Methods

### Ethics statement

Written informed consent was obtained from each of the healthy volunteer participants. The study was approved by the Ethics Committee of the S.Orsola-Malpighi University Hospital of Bologna.

### Samples, libraries construction and sequencing

The mtDNA genomes of 41 Italian individuals previously assigned to haplogroup HV*(xH,V) [17] were selected for sequencing with the Ion PGM^™^ System (Life Technologies, Grand Island, NY, USA) together with two additional individual samples already available at the laboratory of Molecular Anthropology of the University of Bologna. MtDNA libraries were generated by physical fragmentation of two long-range PCR products with Bioruptor sonication system (Diagenode Inc., Denville, USA). After barcoded adapter ligation, libraries were size selected, quantified and subjected to emulsion PCR for template enrichment, being finally sequenced using the Ion PGM Sequencing 200 Kit v2 (Life Technologies, Grand Island, NY, USA). Bioinformatics tools implemented in the Torrent Suite ^™^ 4.0 (Life Technologies, Grand Island, NY, USA) were used with standard settings to process the obtained sequence reads and to align those with high quality scores to the Reconstructed Sapiens Reference Sequence (RSRS) [26] mitochondrial reference genome, as well as to call single nucleotide polymorphisms (SNPs) and small insertion/deletion (INDELs) variants. The sequences recovered have an average coverage of 138.6X (minimum 33X, maximum 361X). Robustness of the applied sequencing method was tested by performing some replicates for randomly selected samples using a traditional Sanger approach and the MitoALL Resequencing kit (Applera, Foster City, CA). All the SNPs and INDELs identified by massively parallel sequencing were confirmed by whole mtDNA Sanger sequencing.

Twelve additional mtDNA genome sequences from HV*(xH,V) individuals belonging to a wider dataset were generated with the following protocol at the facilities of the Tartu Institute of Molecular and Cell Biology and included in the study. Complete mtDNA genomes were amplified in four overlapping fragments. After purification, 48 internal primers were used for sequencing the obtained amplicons using BigDye Terminator v3.1 Cycle Sequencing Kit (Applied Biosystems) on a 3730xl DNA Analyzer (Applied Biosystems). The resulting sequences were aligned and analyzed with the Sequencher v5.0 software (Gene Codes Corporation). All the newly generated sequences are available in GenBank (http://www.ncbi.nlm.nih.gov/genbank/) with accession codes KP340126-KP340180.

The obtained dataset of 55 newly sequenced mtDNA genomes was supplemented with another 15 published HV sequences from a dataset recently deposited in GenBank, but not previously analyzed for their phylogenetic characteristics [41] (accession numbers: JX152993, JX152996, JX153051, JX153057, JX153061, JX153087, JX153134, JX153274, JX153323, JX153371, JX153420, JX153435, JX153443, JX153457, JX153576).

A comparative dataset was then assembled with our consensus sequences and other mtDNA genomes from 246 individuals retrieved from literature, including the Cambridge Reference Sequence (rCRS) and eight non-HV sequences as outgroups, in order to achieve a more precise phylogenetic framing. A multiple alignment procedure was performed using MAFFT v7 (http://mafft.cbrc.jp/alignment/software/) and the obtained output was manually checked with Bioedit (www.mbio.ncsu.edu/BioEdit/bioedit.html) and used as input file for downstream analyses. Details about each sample considered, including the publication reference and geographical origin (when available), are summarized in S1 Table.

### Haplogroup assignment and phylogenetic analyses

A first haplogroup assignment was performed using the HaploFind software [42] which uses PhyloTree Build 16 [28] as underlying classification tree, and manually confirmed or corrected after a parsimonious tree analysis. A second haplogroup assignment, for double-checking, was performed with Mitomaster [43], which makes use of HaploGrep [44] based on PhyloTree Build 16 [28]. The overall HV phylogeny was drawn with the software mtPhyl (https://sites.google.com/site/mtphyl/home), while BioEdit (http://www.mbio.ncsu.edu/bioedit/bioedit.html) was used to visualize the alignment and manually verify the tree topology, tracking the presence of single mutations through the lineages. The mtPhyl software was also used to obtain a first estimate of haplogroup divergences and their error ranges, according to well-established calibration methods [29,45,46].

Phylogenetic trees and Bayesian Skyline Plots (BSPs) were generated with the BEAST package v1.7.2 [47]. The best substitution model was determined using jModelTest v2.1.7 [48]. In order to determine the best clock model and the best tree model, different runs were performed with BEAST and evaluated with a Bayes Factor analysis [49,50]. In respect of the clock model, a strict clock model and an uncorrelated relaxed lognormal (ULN) clock model were compared, while a constant population size model was compared to a Bayesian Skyline model regarding the tree model. The adopted mutation rates were those reported in Soares et al. [30]. For the major monophyletic clades, which have a smaller sample size and an unambiguous phylogeny, we simply used the rate for the entire molecule (1.665 × 10^−8^ substitutions per nucleotide per year). For the whole dataset, which is affected by a heavy load of branch length variation, runs were instead performed with one as well as two partitions, the latter consisting of the coding region, to which we assigned a rate of 1.708 × 10^−8^ substitutions per nucleotide per year and the non-coding region, to which we assigned a rate of 9.883 × 10^−8^ substitutions per nucleotide per year. Two-partition runs were also performed on the entire dataset with imposed monophyly on the major haplogroup branches HV0, HV1, HV4, HV2-73 and HV-16311. The best substitution model determined by jModelTest was TN93+I+G for both the coding and the non-coding part of the alignment. Evaluating the maximum likelihood estimates for the different combination of clock models (i.e. strict clock/ uncorrelated relaxed lognormal (ULN) clock) and tree models (i.e. constant population size/ Bayesian Skyline) using Bayes Factor (BF) analysis [51] revealed a decisive support for a ULN clock (log10(BF) = 12.2) and for the Bayesian Skyline tree model (log10(BF) = 73.7); these parameters were therefore chosen for the analyses. A total of 10–20 million chains were performed for the single lineages, while 50 million chains were executed for the entire sequence set, to get reliable ESS values. Multiple runs were performed for each dataset and combined using logCombiner. The maximum clade credibility was determined using TreeAnnotator and visualized with FigTree (http://tree.bio.ed.ac.uk/software/figtree/). Median-joining networks [52] were computed with Network 4.11 (www.fluxus-engineering.com), excluding positions in the two poly-C stretches. Networks were calculated in two ways: 1) giving equal weights to all nucleotide sites, to show potential conflicts in the reconstructed topology due to back and recurrent mutations; 2) giving weights inversely proportional to the frequency of a given mutation in the whole mtDNA phylogeny, from a minimum of 1 for the most recurrent mutation, to a maximum of 99 for singletons [29], to simplify the topologies with our a priori knowledge about each position. Networks were drawn without applying pre- or post-processing steps and visualized by means of Network Publisher. Branches showing starlike signals of expansions were dated using the rho statistic [53] implemented in the Network software, with the calculator provided in the Soares et al. Supporting Information [29]. Correspondence analysis (CA) on haplogroup frequencies was performed with the R *ca* package [54]. For the distribution of major HV sublineages along the Italian peninsula, the majority of the sampled localities referred to the study of Boattini et al. [17], with the addition of 16 sampled sites screened for the presence of HV*(xH,V) lineages, but for which full mtDNA genomes were not generated.

## Results

### Overall tree topology

**Fig 1:**
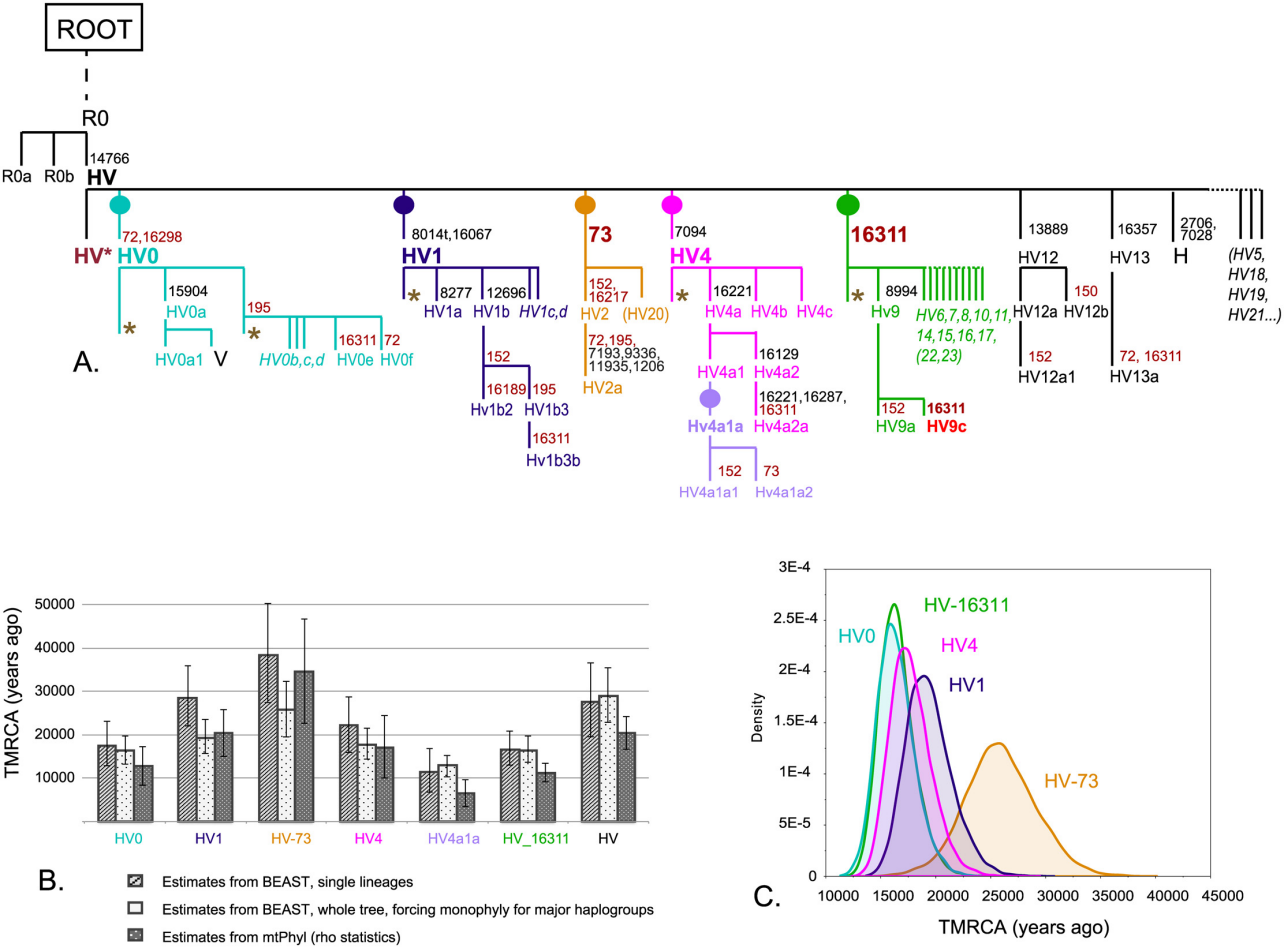
HV phylogeny and dates. **(A)** Schematic tree of the HV phylogeny, with important branches highlighted. Major nodes HV0, HV1, HV-72, HV4 and HV-16311 are marked with different colors. Mutations defining major clades are indicated, as well as mutations recurrent in the dataset, in dark red font. HV* and other lineages with an asterisk indicate positions of the tree for which we find potentially new lineages with our Italian data. The problematic position of HV9c is highlighted. (**B)** TMRCAs for major nodes and for the whole HV tree calculated by BEAST on the single lineages, on the whole dataset with imposing monophyly on major branches, and by mtPhyl. Confidence Intervals (of HPD intervals from BEAST runs) are visualized by error bars. (**C)** Probability estimates of the root height (TMRCA) calculated by BEAST on the imposed monophyly dataset.

The topology of the 316 examined sequences was first explored using mtPhyl, which reconstructs the maximum parsimony phylogenetic tree applying a superimposed topology from PhyloTree and annotating mutations against the rCRS. Sequences are therefore first classified according to the presence/absence of haplogroup diagnostic mutations. The robustness and parsimony of the tree was manually checked against the alignment. The resulting tree highlights mutations previously reported in PhyloTree, as well as new mutations detected in our dataset (S1 Fig). The major monophyletic lineages recognizable within the HV*(xH,V) clade are HV0, HV1, HV2 (which is included in a separate branch with other sequences which share the A73G mutation) and HV4. These lineages account for the majority of the analyzed mtDNA genomes, while other lineages within the haplogroup are represented by a reduced number of individuals. Haplogroups HV6, HV7, HV8, HV9, HV10, HV11, HV14, HV15, HV16 and HV17 share the (highly recurrent) T16311C mutation, being therefore joined under a common node previously recognized and named HV3 [14,55]. Other sporadic lineages within the HV block, which do not belong to any defined branch, are labeled as HV*. The reconstructed topology is summarized in Fig 1A.

The above-described phylogeny was further explored with the help of a BEAST-generated tree (S2 Fig). On a direct comparison, the phylogeny obtained with BEAST does not completely overlap with the one reconstructed with mtPhyl because of three main reasons: 1) the forced dichotomy of the BEAST tree generates weakly supported branches and cannot resolve the subdivision in parallel lineages characteristic of the HV internal splits; 2) the number of private mutations varies extensively between parallel lineages, creating a range of times-to-the-most-recent-common-ancestor (TMRCAs) for haplogroups which split at the same time within the HV branch and suggesting different evolution rate and the presence of a relaxed clock; 3) the recurrent mutations create ambiguous branching.

The first two objections are exemplified in Fig 1B and 1C. While the whole HV set coalesces at 27–29 kya (20.5 kya according to mtPhyl), some sublineages characterized by more mutations than the average show an older coalescence, i.e. the whole HV-73 block, which coalesces at ~38 kya (35 kya according to mtPhyl, 26 kya with imposed phylogeny in BEAST). The other major nodes, in spite of branching simultaneously at the root of HV, also coalesce at different times, such as ~17 kya for HV0, 20–28 kya for HV1, 17–22 kya for HV4 and 11–16.5 kya for the HV-16311 block (See S2 Table for exact TMRCA and CI from BEAST). This variation in coalescence times is found also in the mtPhyl TMRCAs, which may vary from the BEAST estimates because of the different dating method (i.e. the Rho statistics on the average branch distance), but fall within the range of confidence intervals (Fig 1B).

Concerning the point 2), Bayes Factor analysis for the whole dataset revealed a strong support for a relaxed clock: this allows branch length variation between branches. On the contrary, for the single major nodes of the HV*(xH,V) clade, the comparison between relaxed and strict clock does not affect the likelihood significantly. Accordingly, for the single major nodes we applied the strict clock for our BEAST estimates and we preformed runs without the partition of the dataset to simplify our models.

Concerning the point 3), the whole topology reconstructed with BEAST fails to resolve the relative position of certain branches, with nodes characterized by a low posterior probability (details in S1 Text). S3 Fig shows a second BEAST tree generated by imposing monophyly on the major haplogroup branches HV0, HV1, HV4, HV2-73 and HV-16311, and used to extract TMRCA for single lineages (Fig 1B).

### Geographic distribution of major HV*(xH,V) lineages

**Fig 2:**
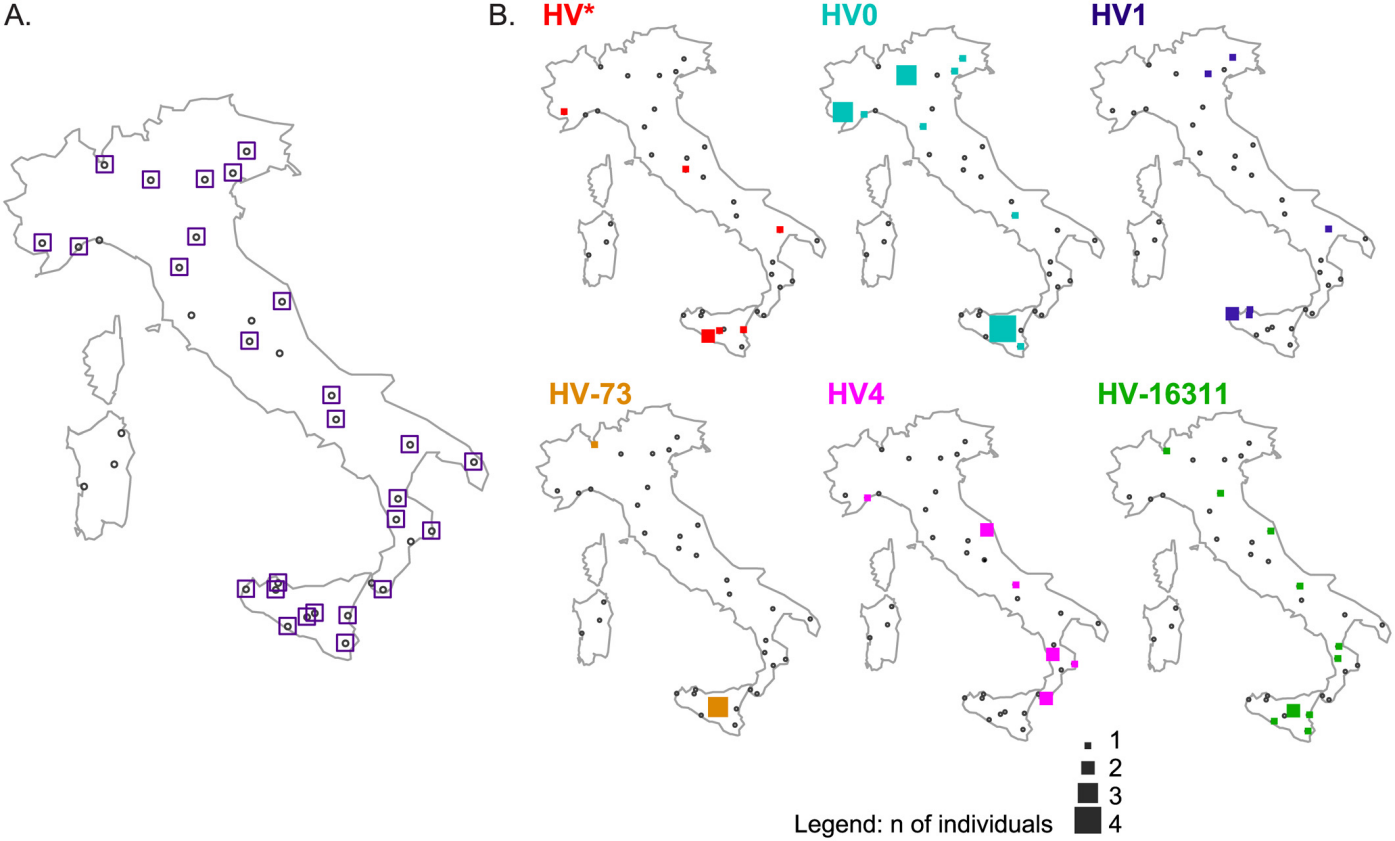
Presence of major HV lineages in Italy. (A) presence of haplogroup HV*(xH,V); (B) presence of major lineages HV0, HV1, HV-73(HV2), HV4, HV-16311. Gray dots indicate sampled sites. The size of the square is proportional to the number of HV individuals in each site (see legend).

Fig 2 localizes the distribution of the major HV*(xH,V) lineages in Italy, with 36 sample sites distributed all over the peninsula and major islands and more than 1,000 individuals screened (S3 Table). Major trends are distinguishable: HV0 appeared to have higher frequencies in Northern Italy, with the exception of Enna, in Sicily. Conversely, HV4, HV* and HV-73 were more frequent in Southern Italy.

An approximate geographic distribution of the most represented lineages in our dataset is visualized by means of a Correspondence Analysis (CA) performed on haplogroup frequencies (S4 Fig). Variation in the distribution of major HV*(xH,V) lineages follows a geographical pattern, with HV0 and HV-16311 samples mostly coming from North/Western Europe and Northern Italy, HV4 individuals originating from Southern Europe (in particular, from the Franco-Cantabrian refugium) and Italy “others” (with this general label we refer to Italian sequences retrieved from literature for which we do not have further geographic information), HV* subjects being sampled from Southern Italy and Eastern Europe (mainly Russia and Baltic countries), HV-73 and HV1 individuals coming from the Middle East (i.e. Turkey, Yemen, former Assyrian), the Caucasus and Africa (mainly Egypt, Tunisia and East Africa). Contrary to the data collected for the Italian peninsula, our comparative dataset is affected by the availability of mtDNA HV genomes, with more data retrieved from previous publications on the Franco-Cantabrian refugium [4], Eastern Europe [14], the Caucasus and the Middle East [15] (see S1 Table). From a previous survey that included haplogroup data for sublineages of HV, HV0 appeared to be more frequent in Northern and Central European populations, as well as in Sardinia and Spain, with overall frequencies ranging from 4.5% to 11% [31].

S1 Fig highlights the position of the Italian individuals within the reconstructed phylogeny. Exclusive Italian lineages, shared by more than one individual, are found within haplogroups HV1, HV4*, HV-16311* and HV0*. The figure also highlights lineages that belong to Eastern Europe, the Caucasus and Middle East. In many cases, Italian isolate lineages are in proximity to Eastern ones (e.g. HV1a1; HV4a2, HV4c and HV4b; HV2a; HV13) and, in two cases, within previously unclassified branches of HV* (e.g. the newly proposed HV18).

### Major lineages within the HV*(xH,V) block

**Fig 3:**
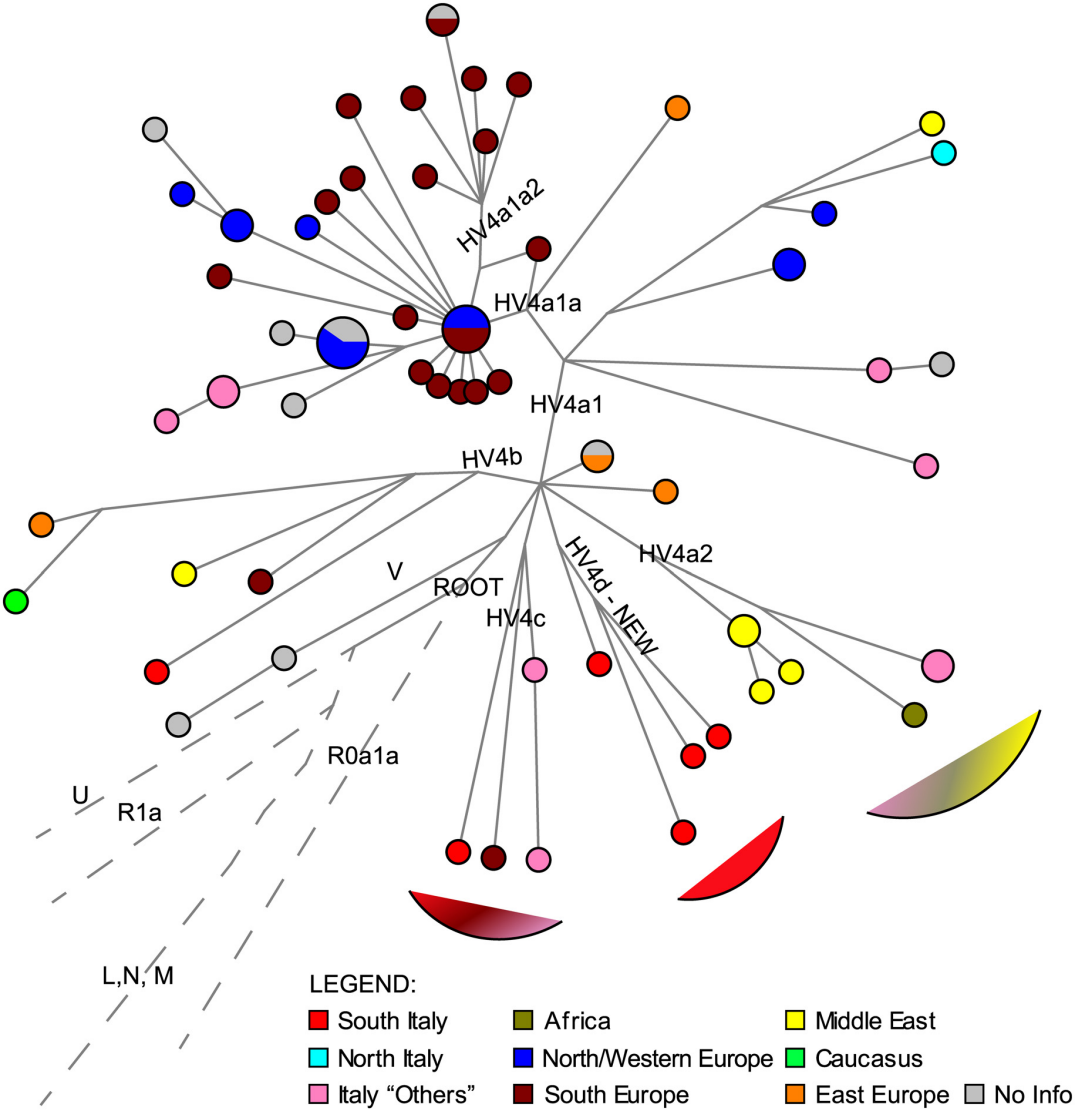
Median Joining network of HV4 lineages. Mutations weighted proportionally to their frequency in the phylogeny.

**HV4** is a clearly monophyletic lineage defined by the stable T7094C mutation. Early divergent sequences are found in provinces from Central-Southern Italy, between HV4c and HV4b in the Network (Fig 3). This branch, defined by two mutations, appears to be endemic and could represent a new branch within the haplogroup (which we tentatively call HV4d, following the PhyloTree nomenclature). HV4b and HV4c branch similarly at the root, with a coalescence time of around 15 kya (or 17 kya according to mtPhyl). Both these lineages include Italian individuals and individuals from eastern regions, Middle East, Caucasus, Eastern Europe. HV4a shows a TMRCA of ~15 kya. The earliest splitting clade, HV4a2, again includes Italian, as well as eastern individuals. HV4a1a is a major sublineage within HV4. It coalesces at ~13 kya and shows a clear signal of expansion which mainly involves the Franco-Cantabrian refugium.

**HV0** is defined by unstable and recurrent mutations within the hypervariable segments: nucleotide position 72 appears to back mutate four times within this branch, according to the topology reconstructed by mtPhyl (S1 Fig), while position 16298 would have back mutated twice. The branching of these mutations appears therefore particularly unstable and difficult to reconstruct with the network, which indeed is very reticulated at its core (see S6 Fig). Reticulation at the root still persists also when the recurrent mutations are downweighted (S7 Fig). The main monophyletic branch within HV0 is HV0a (from which, in turn, haplogroup V stems). HV0a coalesces at around 12.5 kya in the BEAST tree and at 11 kya in the mtPhyl one.

**HV1** is another subclade well represented in the studied dataset. Sublineages HV1a, HV1b, HV1c and HV1d are clearly distinguished by mutations in the coding region, with HV1a and HV1b representing distinct branches in the network, even when positions are given equal weight (S8 and S9 Figs). A small expansion within HV1b is geographically localized in Eastern Europe (S8 and S9 Figs). One branch at the root of this haplogroup, defined by a stable coding mutation, is represented by three Italian individuals and could indicate another endemic sub-branch, here named HV1e (S1 Fig)

The unstable A73G! back mutation within HV characterizes a group of lineages including the major branch **HV2**. HV2 constantly appears as the earliest split of the HV phylogeny in our BEAST trees, probably because of its high number of mutations that differentiate it from the rest of the tree. An early branch within this haplogroup includes Italian individuals and a subject from India, again highlighting a potentially eastern component for HV variation observed along the Italian peninsula (S10 and S11 Figs). The HV2 branch coalesces at ~16 kya according to BEAST and 26.15 kya according to mtPhyl.

**HV12** is another subclade whose monophyly is revealed by the BEAST tree in S3 Fig. This branch, found in our dataset in Turkey, Caucasus and India, coalesces at 21.31 kya according to the mtPhyl reconstruction, and at 20 kya in the BEAST tree.

Within HV, an internal clade is defined by the mutation **T16311C!**. This branch includes HV6-HV11 and HV14-HV17. The recurrence of 16311 in the dataset is the cause of reticulations (S12 Fig); it is in fact found also as a characterizing mutation for HV0e, HV4a2a, and it is found independently in five other individuals (within HV0d, HV1b3, HV4a1a4, HV-73 and HV1c). Nevertheless, the unity of this block is recognized by the generated BEAST trees (S2 Fig) and by the network (S12 Fig), except when recurrent mutations are downweighted (S13 Fig). The HV14, HV15, HV16 and HV17 sequences were represented by a predominance of North/Western Europeans, while the HV6, HV7, HV8, HV9, HV10 and HV11 by a predominance of North/Western and Eastern Europeans (S14 Fig). Our samples from the Italian dataset generally fall outside these previously labeled haplogroups. In fact, we also find the T16311C! mutation in a number of individuals previously defined as HV*, which include other subjects from the literature belonging to the Italian and Eastern European populations. Each of these lineages is further defined by private mutations (both coding and non-coding; another characteristic southern Italian cluster is defined by recurrent mutation at position 310). We therefore assume that additional branches can be discovered within the HV-T16311C! block and named accordingly.

A deeper phylogenetic analysis of these lineages and their characterizing mutations is described in more detail in S1 Text, while related TMRCAs are reported in S3 Fig.

### Time frame and expansion

**Fig 4:**
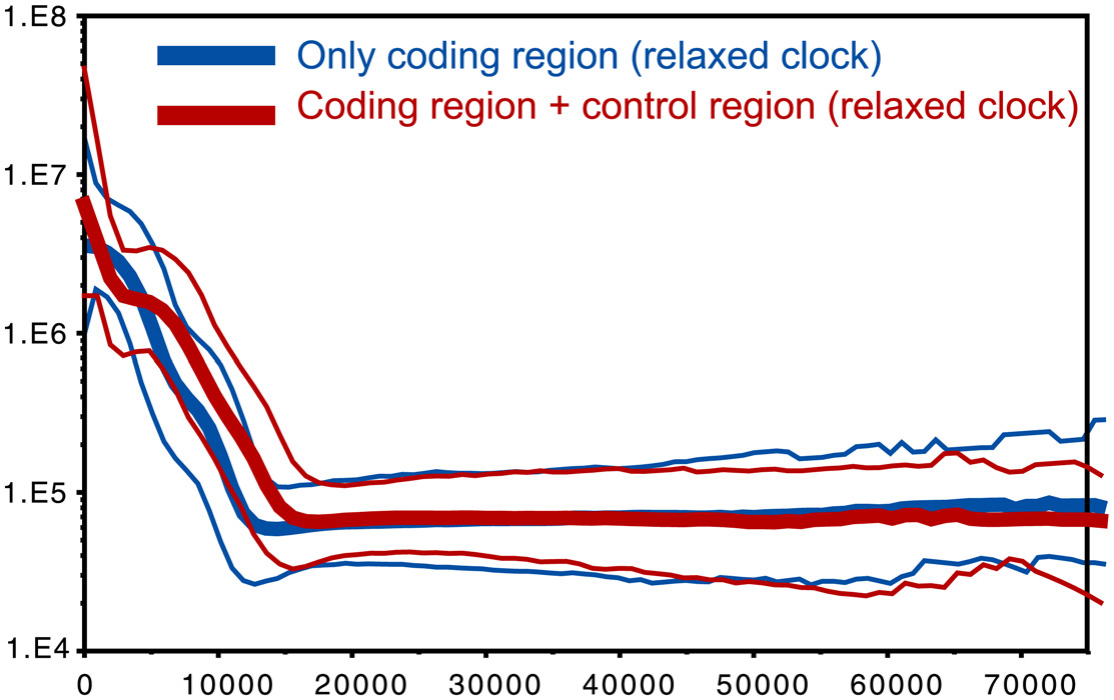
Bayesian Skyline plot of the whole HV dataset, with the coding region only and with the full molecule (see legend).

In general, age estimates obtained from the coding region only by applying the appropriate rates from Soares et al. [29] are lower than those obtained from the full sequence. The strong expansion effect reflected in several HV subclades probably led to a recent accumulation of mutations that were less subjected to purifying selection, simulating the effect of an accelerated rate of evolution [29,56,57]. Therefore, we performed runs for the whole dataset (as in S2 Fig) with two partitions and two different rates, one for the coding and one for the non-coding segment, following the method used in Lippold et al. [58]. The overall signal of expansion visible in the BSP (Fig 4) shifts from 15 kya (full sequence) to 12 kya (coding region only). In all cases, the bigger expansion impact in Eurasia seems to have happened in a pre-Neolithic time.

Within the examined dataset we identify lineages with more recent coalescence times (and in particular with more recent expansion times), which are compatible with Neolithic events and, in some cases, appear to be restricted to specific geographical regions (see S1 Fig and the starlike pattern in S8 Fig). The BPS plots obtained for only the HV individuals with and without partitioning the dataset, as well as the BSP plots for single lineages, are shown in S15 Fig. Haplogroup HV0 shows a steep constant expansion that starts at its coalescence. Haplogroup HV1, with an older coalescent time, also displays a clear expansion, with an increase at around 6 kya. This signal was broken down into the two major lineages within HV1, HV1a and HV1b, to highlight the recent expansion experienced by HV1b (starting at ~3 kya) that is visible also in the network (S8 Fig). HV1a, on the other hand, shows an older expansion starting around 10 kya. The BSP for the whole HV4 haplogroup indicates an increase which starts at ~7 kya. The expansion within HV4a1a, clearly visible in the network (Fig 3) and dated with the rho statistic at 7.5 kya, appears less steep in the corresponding BSP. The HV-16311 block shows a steeper expansion at the root. Finally, the HV-73 block started to expand at ~17 kya, after its coalescence.

## Discussion

The present study explored mtDNA variation patterns within HV*(xH,V), a haplogroup of pre-Neolithic origin present at relatively low frequencies in current Eurasian populations. We particularly focused on its distribution across the Italian peninsula, since this haplogroup was found to be the one with the oldest TMRCA in a previous fine-scale survey of the variability of uniparental markers in the Italian population [17]. The 55 whole mtDNA genomes sequenced in the present study were contextualized within the Eurasian mitochondrial landscape by integrating them in a broad dataset, which included individuals retrieved from literature for a total of 316 complete mtDNA sequences. This enabled us to both revisit the phylogeography of the HV* sublineages and explore the characteristic pre-Neolithic signals emerged from the analysis of Italian mtDNAs.

The first effort was spent to reconstruct the HV*(xH,V) phylogeny. The obtained topology appears to be uncertain due to the presence of recurrent (and therefore unstable) mutations located in the hypervariable segments, which show up multiple times in the tree as defining for different sub-branches (Fig 1A and S1 Fig). The simultaneous radiation of many parallel lineages within HV in a short time interval (due to a rapid population expansion) creates consistent problems in the reconstruction of a tree according to the approach implemented in the BEAST software (S2 Fig). In fact, recurrent mutations made some nodes unresolvable and also produced a high level of reticulation in the networks, which in some cases could not be eluded even when the recurrent sites were downweighted (see S7 and S13 Figs). In spite of the instability of some of the lineage-defining mutations, major blocks are recognized by different methods with consistency, such as the clades HV0, HV1, HV4, the HV-16311 block, which was previously designated as HV3 [14,55] and, more tentatively, the HV-73 block (with a few exceptions of individuals that appeared unclassifiable). The monophyly of HV-16311 (or HV3) was previously considered uncertain due to the recognized instability of the 16311 mutation [59,60], although our full mtDNA genome analysis seems to confirm the robustness of the block (S2 and S12 Figs). On the other hand, we suggest a cautious approach when assigning HV sub-haplogroups to samples, especially if only information deriving from the hypervariable segment(s) is available. Both previous haplogroup assignments reported in literature and our automatic haplogroup assignment performed with a dedicated software (i.e. HaploFind and Mitomaster) had to be manually checked for consistency with the reconstructed phylogeny and, in some cases, entailed the complete re-assignment of the studied individuals to different haplogroups (marked in S1 Table and in S1 Fig). Wrong assignment was indeed observed for full sequence data within HV0f, HV9c and with the HV-16311* block. For this reason, the position of some individual sequence has to be carefully traced through the tree, bearing the PhyloTree reference in mind, and the incidence of recurrent mutations. In general, we realize that strong claims on the origin and diffusion of specific lineages should be avoided especially in less certain parts of the topology, as well as when only hypervariable segment(s) data are available and if a careful reconstruction of the phylogeny for proper haplogroup assignment is not performed.

Demographic reconstructions (Fig 4) highlight a major signal of expansion from pre-Neolithic times. This strong radiation probably boosted the accumulation of mutations, creating, in some cases, a higher perceived evolutionary rate [29,57]. Parallel branches radiating from the same node apparently coalesce at different times (Fig 1B and 1C). An alternative explanation for such rate heterogeneity is positive selection having acted/acting on some of the variants characterizing the examined sequences. In fact, in the earliest diverging lineage (HV-73) we observe one non-synonymous mutation, namely A9336G (rs28474779), which causes an amino-acid change from Met to Val in the *COX3* gene, which encodes the terminal component of the respiratory chain involved in the aerobic production of energy [61]. However, functional predictions for this substitution suggest that it most likely lacks any phenotypic effect (SIFT score = 0.58 and Polyphen PSIC = 0) [62, 63], being thus an unlikely target of natural selection. Moreover, this mutation is also present in the L1c1a2a haplogroup (according to PhyloTree Build 16, [28]), which is a typical Pygmy lineage [64], so that it is not clear which selective pressure would have acted on it in these highly differentiated populations.

The massive simultaneous branch radiation produced star-like diffusion patterns visible in the networks, which lack any core lineage that could indicate from where they have spread, and generated low posterior probability for the nodes of the BEAST trees (S2 Fig). By applying the mutation rate reported in Soares et al. [29] and a relaxed clock model on coding and non-coding mtDNA partitions, we obtain a coalescent time for the HV cluster of ~27–29 kya, which appears to be older than the mtPhyl estimate, but in the same range when considering the CI of the two dates (Fig 1B). Our TMRCAs are thus compatible with the ones proposed by Zheng et al. [65], which similarly found an expansion in BSPs ranging from 21 ky to 28 kya. The new faster rate proposed by Rieux et al. [57] would probably produce younger coalescence times, but those would still be in line with a pre-Neolithic major radiation. This radiation could also be associated with a repopulation event that began after the last glacial acme. From an archaeological perspective, during this time European populations experienced a radical change in their material culture, with the decline of the Gravettian culture and the diffusion of the Madgalenian one from the Franco-Cantabrian region, which spread until Portugal and Poland, but did not succeed in crossing the Alps. On the other hand, the Italian peninsula experienced a completely different scenario, with the Gravettian culture shifting toward another similar culture, the Epigravettian, which is characteristically found in southern areas [66,67]. This divide between the main European and Italian Mesolithic cultures could be thus correlated with the phylogeographic distribution of mtDNA haplogroup HV, and with the presence of deeply divergent lineages in Italy.

The major discovery of our analysis is the ancient divergence and isolation of the Italian HV* lineages. New sources of variation are found within HV4 (mostly in Southern Italy) and HV0 (mostly in Northern Italy), as well as within HV* and HV-16311*. Exclusive Italian lineages splitting at the root of the major branches can be assigned to new autochthonous haplogroups, such as the proposed HV4d, HV1e, HV0g. These autochthonous early diverging lineages may indicate an ancient local presence, but we cannot dismiss the hypothesis that they all arrived in the peninsula with historical migrations. Another interesting signal comes from Italian isolate lineages that are in proximity to those found eastwards (e.g. from Middle East, Caucasus and Eastern Europe, see S1 Fig, Fig 3 and S8, S9, S10, S11 Figs). This connection, even if based solely on the maternal perspective, suggests that Southern Italy could have been involved in the early crossing of people coming from the east. Nevertheless, this does not exclude that more data from the rest of Europe could reveal similar signals of early ancestry. If the Mesolithic specimen found in Favignana (Sicily) belonging to mtDNA haplogroup HV1 [18] will be confirmed, this may prove the antiquity of this haplogroup in Southern Italy. We are aware that these data were generated with PCR-based methods, and therefore do not meet the golden standards for authentication of aDNA results (i.e, for the estimate of contamination and damage patterns) [68]. Our phylogenetic reconstruction indicates that some lineages within haplogroup HV, which was previously included as a whole in the “mitochondrial Neolithic package” as characteristic marker of the Linear Pottery culture (LBK) in central Europe [13], might have left traces of an early diffusion also in southern regions, albeit we surely cannot pinpoint if this early diverging lineages would have arrived before or after the Neolithic diffusion. Up to date, the major release of genomic and mtDNA data for Europe is centered on sites from central and northern Europe, northern Spain, and Asia (i.e. Caucasus and central Asia) [13, 33, 69]. The few data available for Italian sites report haplogroup R* for the most ancient site [70], basal lineages of U, the predominant Paleolithic lineage [13, 20] and lineages within H, J1 and X2c in more recent times [13, 69]. Only the release of high-quality NGS aDNA data from specimens belonging to Southern Europe, and especially Italy, can contribute to elucidate the time and the modality of arrival of this particular lineage in the Mediterranean area.

The deep phylogenetic structure within HV*(xH,V), reflected by the presence of long separated branches, is similarly found in other species studied as biological markers of the post glacial re-colonization from southern refugia [38]. The isolation of Southern Italy and the presence of highly heterogeneous environments encapsulated within mountains could have created an effect of multiple refugia [71–73]. While other glacial refugia played a major role in the recolonization of the more northern parts of the continent, leaving a genetic signature represented by clear starlike expansion patterns (see haplogroup HV4 and the role of the Franco-Cantabrian refugium, [4,9,32]), Italy could have experienced an appreciably different prehistory, characterized by isolation and a major effect of the subsequent migratory flows through the Mediterranean basin [12]. What we find in Italy (and particularly in Southern Italy) might be the relict of a substructure that was subsequently reduced after the Neolithic expansion and the other population movements culminating into the major Bronze Age migrations [69,74,75]. In this case, we might not have identified a proper center of expansion and re-colonization, but more likely a reservoir of ancient variability.

Summarizing, the early time of coalescence and expansion, the presence of autochthonous divergent branches linked to the east and the differences between the patterns of diversity found in Italy and those found in other European regions like northern Spain/France make it difficult to reconcile the observed HV variation in Italy with early Neolithic or later Bronze Age influences alone. Nevertheless, we cannot completely exclude the effect of subsequent expansion events: in fact, in the absence of more fine-grained data for this particular lineage from Europe and Asia, we should not entirely reject the hypothesis of a recent introduction.

Finally, our data allow us to review the origin and spread of haplogroup HV4. Gómez-Carballa et al. [4] highlighted a recent presence of this lineage in Italy, possibly coming from the historical contact with the Spanish contingents, but did not exclude an earlier diffusion of the haplogroup in the Mediterranean basin. We provide evidence for their intuition, describing the concomitant presence of deeply diverging HV4 lineages in Italy and in the Near East. This suggests that the haplogroup reached the Southern Italian refugia earlier than the Franco-Cantabrian one. Therefore, HV4 could be considered as a characteristic Italian pre-Neolithic lineage, together with the previously suggested haplogroup U5b3 [76].

In conclusion, with our analysis we stress the importance of a careful phylogeographic analysis, the resolution of which can be considerably improved by the analysis of full mtDNA genomes rather than its hypervariable segment(s) only. Haplogroup HV, with its peculiar topology made up of an array of parallel branches and including several recurrent mutations, poses challenges in topology reconstruction and, therefore, haplogroup assignment. We also discuss the role of Italy as a glacial refugium for the human species, interpreting the observed numerous long isolated lineages as a relic effect of a more complex pre-Neolithic structure. These results are important to understand population movements occurred during the Mesolithic/Early Neolithic and will greatly benefit from the inclusion of larger datasets from previously understudied key geographical regions. In particular, more insights are expected from areas that probably acted as a crossroad during the initial radiation of the first European colonization, such as Western and Central Asia. Finally, the release of more aDNA data from southern Italian sites could integrate our perspective on the early population movements that encompassed the whole European continent.

## Supporting Information

S1 Fig Phylogenetic tree of the HV sequences of our dataset, generated with mtPhyl and manually checked for consistency and parsimony. The tree includes the mutations characteristic of each branch as well as information on the geographic origin of the samples (if they come from Italy or from eastern regions) and on cases where the topology had to be manually corrected.
(XLSX)

S2 Fig Phylogenetic tree with all the 316 sequences of the dataset Realized in BEAST. (PDF)

S3 Fig Phylogenetic tree with all the 316 sequences of the dataset. Realized in BEAST, after imposing monophyly of major haplogroups branches HV0, HV1, HV4, HV-73 and HV-16311.
(PDF)

S4 Fig CA analysis of haplogroup frequencies (major nodes) for geographic areas present in the dataset. (PDF)

S5 Fig Median-joining networks for major lineage blocks: Haplogroup HV4. Mutations are given equal weight.
(PDF)

S6 Fig Median-joining networks for major lineage blocks: Haplogroup HV0. Mutations are given equal weight.
(PDF)

S7 Fig Median-joining networks for major lineage blocks: Haplogroup HV0. Mutations weighted proportionally to their frequency in the phylogeny.
(PDF)

S8 Fig Median-joining networks for major lineage blocks: haplogroup HV1. Mutations are given equal weight.
(PDF)

S9 Fig Median-joining networks for major lineage blocks: haplogroup HV1. Mutations weighted proportionally to their frequency in the phylogeny.
(PDF)

S10 Fig Median-joining networks for major lineage blocks: haplogroup HV-73, HV5, HV12, HV13 an HV*. Mutations are given equal weight.
(PDF)

S11 Fig Median-joining networks for major lineage blocks: haplogroup HV-73, HV5, HV12, HV13 an HV*. Mutations weighted proportionally to their frequency in the phylogeny.
(PDF)

S12 Fig Median-joining networks for major lineage blocks: haplogroups within the 16311 block, including HV-16311* and HV*. Colored by haplogroup affiliation. Mutations are given equal weight.
(PDF)

S13 Fig Median-joining networks for major lineage blocks: haplogroups within the 16311 block, including HV-16311* and HV*. Colored by haplogroup affiliation. Mutations weighted proportionally to their frequency in the phylogeny.
(PDF)

S14 Fig Median-joining networks for major lineage blocks: haplogroups within the 16311 block, including HV-16311* and HV*. Colored by geographic origin of the samples. Mutations are given equal weight.
(PDF)

S15 Fig Bayesian Skyline Plots fo the whole HV sequences and for major lineages within HV. X axis: time in years ago. Y axis: effective population size per generation time. The run for the whole haplogroup HV*(xH, V) is performed with the with and without a partition in coding and non-coding and a relaxed clock, the other runs for single lineages are performed with a strick clock and no partition (see Methods for details).
(PDF)

S1 Table List of samples considered in the study with relative information. (XLS)

S2 Table TMRCAs for major haplogroups and for the whole HV branch. (XLS)

S3 Table Complete list of sampling sites and number of HV individuals per site. (XLS)

S1 Text Details on the phylogeny reconstruction. (DOC)
