# Supplementary figures and images for: Fine Dissection of Human Mitochondrial DNA Haplogroup HV Lineages Reveals Paleolithic Signatures from European Glacial Refugia
Source: PLoS One. 2015 Dec 7;10(12):e0144391. doi: 10.1371/journal.pone.0144391 (PMC4671665; doi:10.1371/journal.pone.0144391)

**S2 Fig. Phylogenetic tree with all the 316 sequences of the dataset.**  
Realized in BEAST.

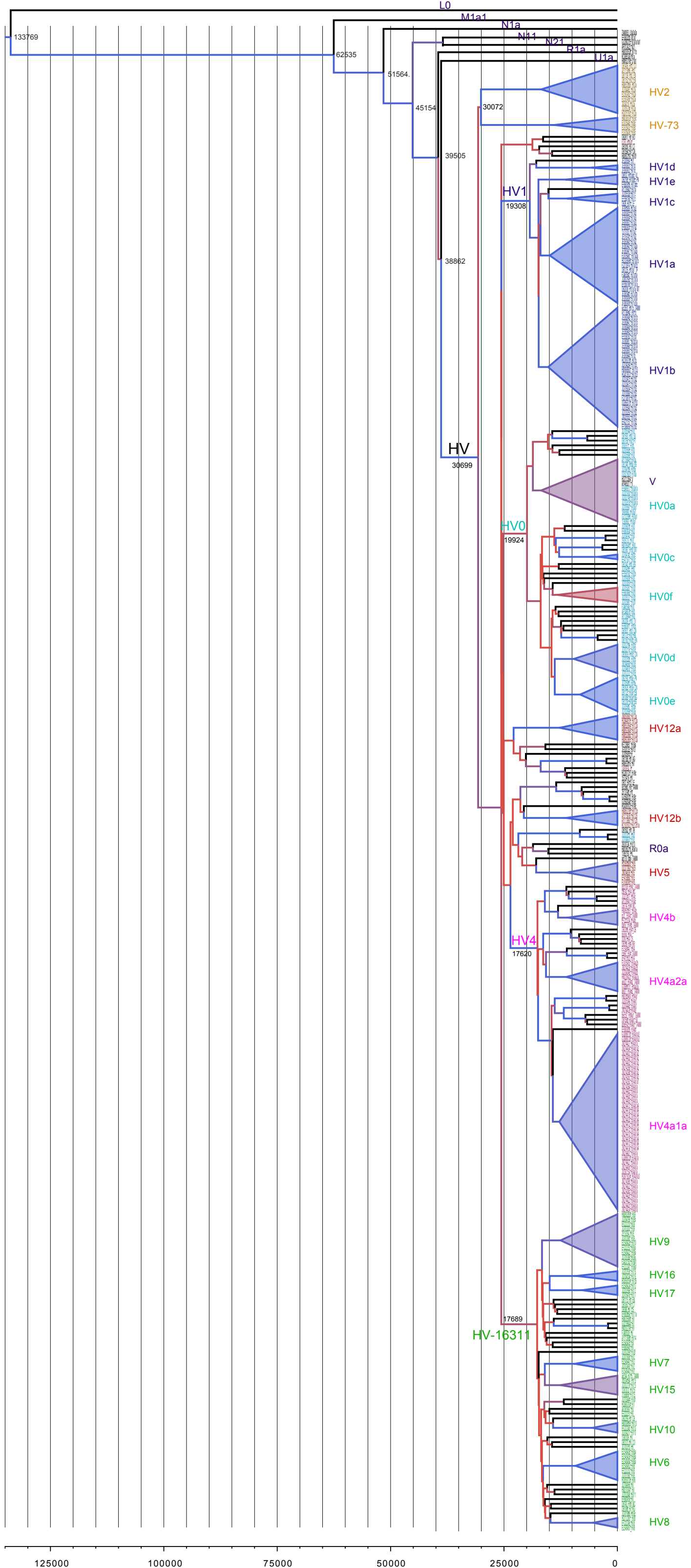

Supplement: S2 Fig — (PDF) [file pone.0144391.s002.pdf]
