# Supplementary material for: Fine Dissection of Human Mitochondrial DNA Haplogroup HV Lineages Reveals Paleolithic Signatures from European Glacial Refugia
Source: PLoS One. 2015 Dec 7;10(12):e0144391. doi: 10.1371/journal.pone.0144391 (PMC4671665; doi:10.1371/journal.pone.0144391)

S4 Fig. CA analysis of haplogroup (major nodes) frequencies for geographic areas present in the dataset.

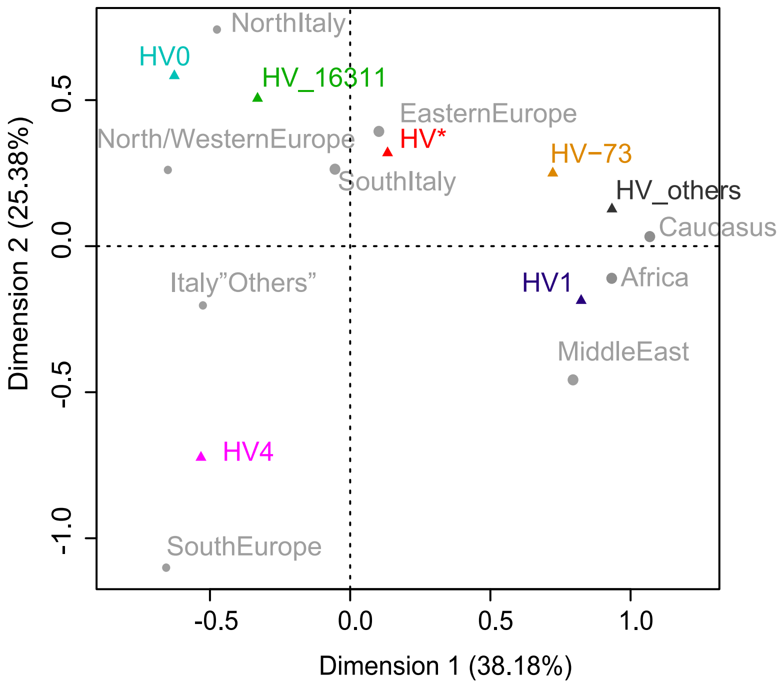

Supplement: S4 Fig — (PDF) [file pone.0144391.s004.pdf]
