# Supplementary material for: Fine Dissection of Human Mitochondrial DNA Haplogroup HV Lineages Reveals Paleolithic Signatures from European Glacial Refugia
Source: PLoS One. 2015 Dec 7;10(12):e0144391. doi: 10.1371/journal.pone.0144391 (PMC4671665; doi:10.1371/journal.pone.0144391)

S9 Fig. Median-joining networks for major lineage blocks: haplogroup HV1.
Mutations weighted proportionally to their frequency in the phylogeny.

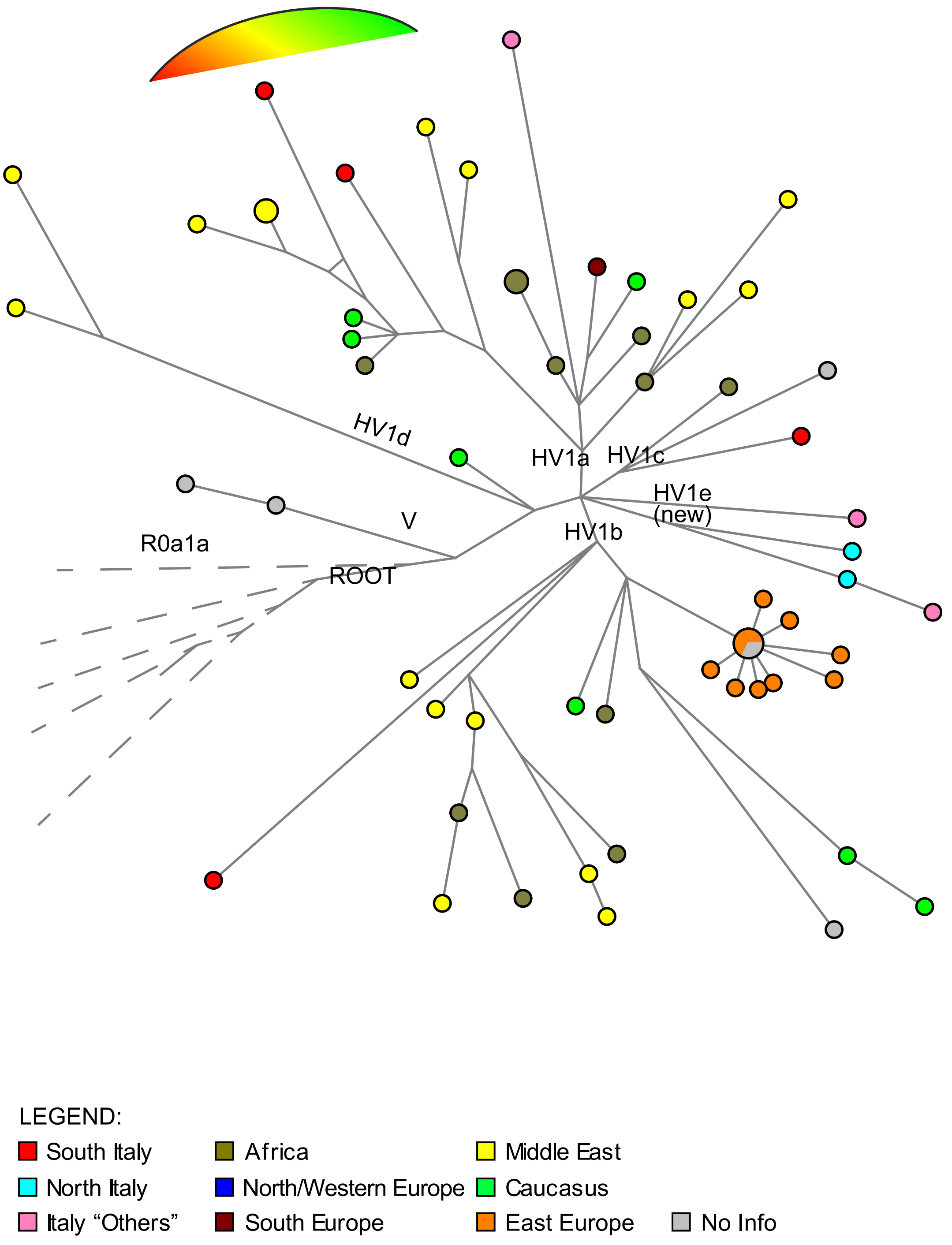

Supplement: S9 Fig — Mutations weighted proportionally to their frequency in the phylogeny. (PDF) [file pone.0144391.s009.pdf]
