# Supplementary material for: Fine Dissection of Human Mitochondrial DNA Haplogroup HV Lineages Reveals Paleolithic Signatures from European Glacial Refugia
Source: PLoS One. 2015 Dec 7;10(12):e0144391. doi: 10.1371/journal.pone.0144391 (PMC4671665; doi:10.1371/journal.pone.0144391)

S10 Fig. Median-joining networks for major lineage blocks: haplogroup HV-73, HV5, HV12, HV13, HV18 and HV*.

Mutations are given equal weights

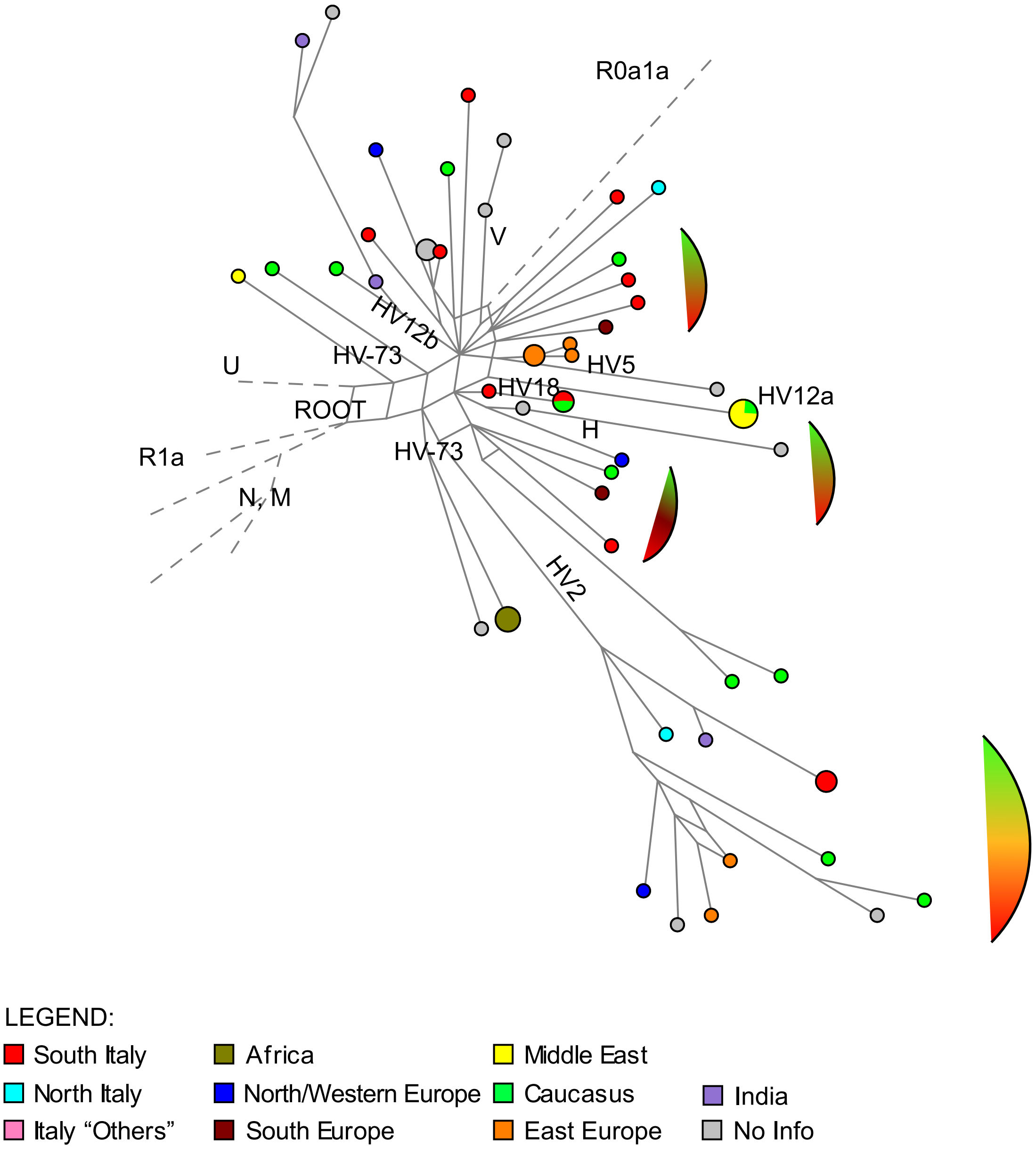

Supplement: S10 Fig — Mutations are given equal weight. (PDF) [file pone.0144391.s010.pdf]
