# Supplementary material for: Fine Dissection of Human Mitochondrial DNA Haplogroup HV Lineages Reveals Paleolithic Signatures from European Glacial Refugia
Source: PLoS One. 2015 Dec 7;10(12):e0144391. doi: 10.1371/journal.pone.0144391 (PMC4671665; doi:10.1371/journal.pone.0144391)

S11 Fig. Median-joining networks for major lineage blocks: haplogroup HV-73, HV5, HV12, HV13, HV18 and HV*.
Mutations weighted proportionally to their frequency in the phylogeny.

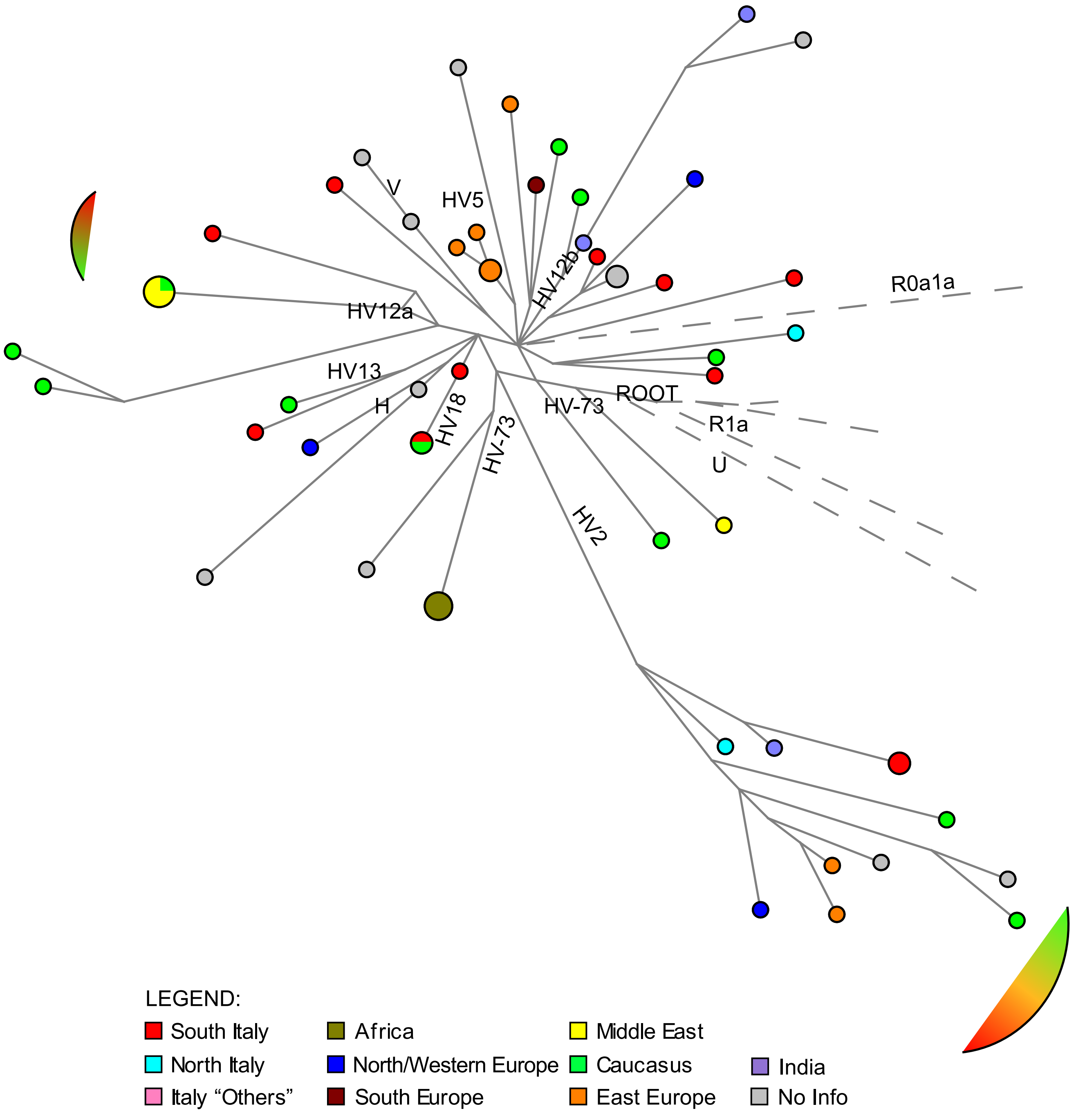

Supplement: S11 Fig — Mutations weighted proportionally to their frequency in the phylogeny. (PDF) [file pone.0144391.s011.pdf]
