# Supplementary material for: Fine Dissection of Human Mitochondrial DNA Haplogroup HV Lineages Reveals Paleolithic Signatures from European Glacial Refugia
Source: PLoS One. 2015 Dec 7;10(12):e0144391. doi: 10.1371/journal.pone.0144391 (PMC4671665; doi:10.1371/journal.pone.0144391)

S13 Fig. Median-joining networks for major lineage blocks: haplogroups within the 16311 block, including HV-16311* and HV*.
Colored by haplogroup affiliation. Mutations weighted proportionally to their frequency in the phylogeny.

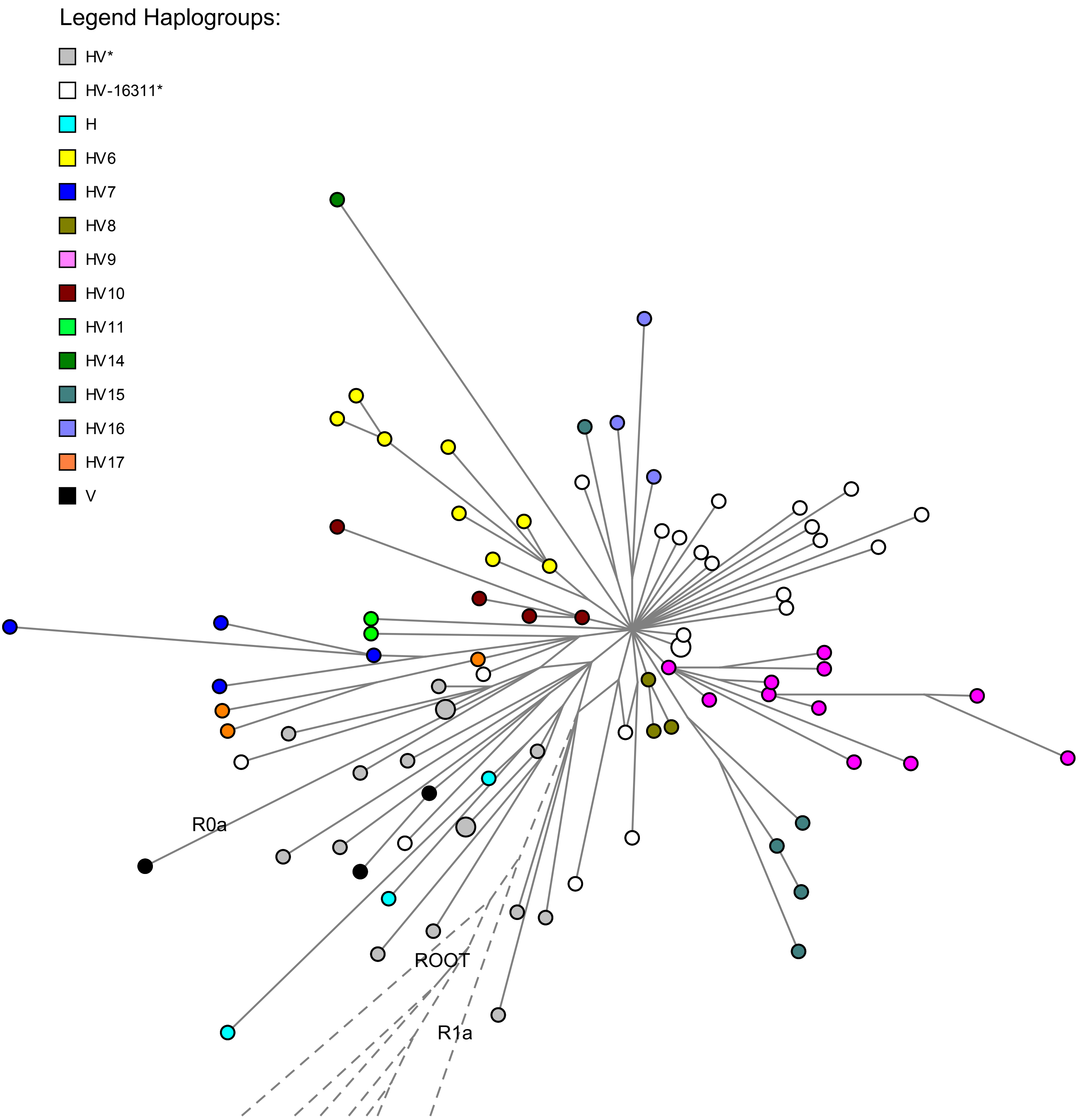

Supplement: S13 Fig — Colored by haplogroup affiliation. Mutations weighted proportionally to their frequency in the phylogeny. (PDF) [file pone.0144391.s013.pdf]
