# Supplementary material for: Fine Dissection of Human Mitochondrial DNA Haplogroup HV Lineages Reveals Paleolithic Signatures from European Glacial Refugia
Source: PLoS One. 2015 Dec 7;10(12):e0144391. doi: 10.1371/journal.pone.0144391 (PMC4671665; doi:10.1371/journal.pone.0144391)

S15 Fig. Bayesian Skyline Plots fo the whole HV sequences and for major lineages within HV. X axis: time in years ago. Y axis: effective population size per generation time. The run for the whole haplogroup HV*(xH, V) is performed with the with and without a partition in coding and non-coding and a relaxed clock, the other runs for single lineages are performed with a strick clock and no partition (see Methods for details).

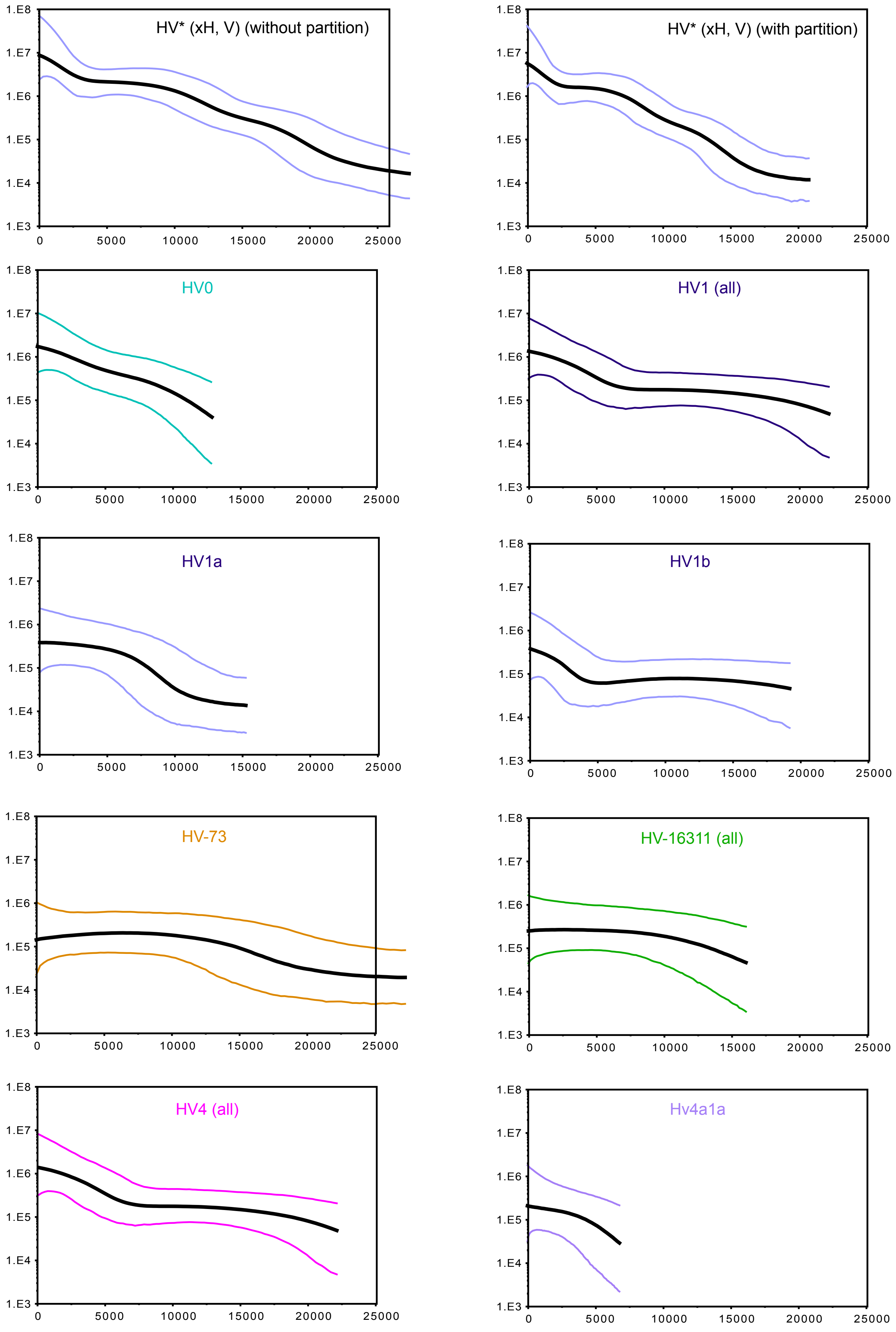

Supplement: S15 Fig — X axis: time in years ago. Y axis: effective population size per generation time. The run for the whole haplogroup HV*(xH, V) is performed with the with and without a partition in coding and non-coding and a relaxed clock, the other runs for single lineages are performed with a strick clock and no partition (see Methods for details). (PDF) [file pone.0144391.s015.pdf]
