# Supplementary material for: Fine Dissection of Human Mitochondrial DNA Haplogroup HV Lineages Reveals Paleolithic Signatures from European Glacial Refugia
Source: PLoS One. 2015 Dec 7;10(12):e0144391. doi: 10.1371/journal.pone.0144391 (PMC4671665; doi:10.1371/journal.pone.0144391)
Supplement: S1 Text — (DOC) [file pone.0144391.s019.doc]

**S1 TEXT – “Fine dissection of human mitochondrial DNA haplogroup HV lineages reveals Paleolithic signatures from European glacial refugia “**

**Unresolved nodes in BEAST**

In S2 Fig. the position of the following branches is not placed coherently: the three HV13 individuals included in the dataset do not form a monophyletic branch; R0a is placed as a subbranch rather than a sister branch of HV, tentatively near other HV13 individuals and close to the HV5 branch (this is probably due to the G73A mutation characterizing R0, which is, however, extremely recurrent in this tree, as mentioned before). Two individuals from the HV-73 block are displaced in the HV* and in the HV-16311 blocks, respectively, and since the latter of the two harbors the 16311 mutation its classification remains unresolvable. Two HV0 individuals with a C16298T! back-mutation are also displaced in a separate branch, and finally, HV12 does not appear to be monophyletic, with its two sublineages being separated in the tree. Apart from these exceptions, the BEAST-obtained tree supports the unity of the HV-16311 and HV-73 blocks, as well as of HV0, HV1 and HV4, while also placing the HV* and the other HV lineages (i.e. HV12, HV13, HV5) at a similar branching level.

The BEAST tree in S3 Fig., generated with the forced monophyly of major haplogroup branches emerging from mtPhyl, displays a coherent phylogeny with HV12 as a monophyletic clade.

**Detailed description of HV lineages**

**HV4** is defined by the mutation T7094C. The variability of this lineage was investigated within a wider panel of European individuals, with a major representation of Eastern Europe, to spot the precise distribution of its sublineages and to confirm the peculiarity of the signal coming from the Italian peninsula. Ancestral sequences, which were automatically assigned to a generic HV4*, are found in provinces from Central-Southern Italy (Macerata, Campobasso, Crotone, Cosenza). In particular, four sequences characterized by the mutation 15514 and the transversion 16192a represent a new potentially endemic lineage that would be named HV4d, by following the hierarchical and alphabetical order of the preexisting nomenclature. One Italian individual from Macerata branches together with other HV4b individuals, sharing only the C16069T mutation, but not the other characterizing C1715T, suggesting an earlier divergence and a high diversity for this sub-branch. The other HV4b sequences of our dataset come from Turkey, Russia, the Caucasus and Greece, displaying a wide eastwards geographic diffusion. HV4c could be similarly defined by the C10356T mutation only and would include also another individual from Central Italy, one from Moldova, and other two Italians who have the defining mutations G2758A and G3834A. Finally, one Russian individual appears alone in a separate branch, similarly ancestral to HV4a. The above-described non-HV4a branches coalesce at around 16 kya (earlier than HV4a1a) according to the whole BEAST tree.

**HV4a** is characterized by the C16221T hypervariable mutation. Its coalescence dates back to 15 kya. HV4a2 is a separate branch characterized by G7805A and the hypervariable G16129A, which back-mutates. This branch is characteristic of Middle East, being observed for one Assyrian and one Jordanian individual, as well as for one Italian and one Egyptian individual. It coalesces at 10 kya ago. HV4a1, identified by the C13680T mutation, includes separate lineages with private mutations represented by two Italians, two French, and one branch with a Balkan and an Iranian individual, together with an individual from Northern Italy sequenced in the present study, which share mutations 8260 and 11440: this branch is here named HV4a1b. Again, the composition of sparse lineages at the root of major branches includes Italian individuals, as well as a more Eastern component. HV4a1a is defined by the T9950C mutation. It coalesces at ~13 kya in S3 Fig. (6.5 kya according to mtPhyl) and in the network shows a starlike pattern indicative of expansion. This expansion was found to be characteristic of both Southern and Nort/Western Europe (i.e. Spain and France – the Franco Cantabrian refugium extensively sampled by Gomez-Carballa et al. [1]), but includes also Italian and British individuals, found in the branch HV4a1a4 and showing the T4452C mutation. The time of the expansion is dated with the Rho statistics and the calculator of Soares et al. [2] at 7.2 kya (CI 4.5-9.9).

**HV0** is defined by the hypervariable mutations T72C and T16298C, which are highly recurrent in the examined dataset. Around 17% of our HV0 sequences come from Northern Italy, while 8% come from Southern Italy and 10% from North/Western Europe. HV0a is defined by the mutation C15904T. A sister lineage defined by the back-mutation T195C! contains lineages previously assigned to HV0*, as well as to HV0b, HV0c, HV0d, HV0e, HV0f. This block is partially recognized in the Network, in which HV0d clusters with previously unassigned lineages, but only when equal weights are given (S6 Fig.). The 195 block disappears when recurrent positions are downweighted (S7 Fig.). HV0f poses problems in the individual assignment because it is characterized by the recurrent back-mutation C72T!, and some individuals have to be reassigned to other branches manually (indicated in S1 Fig.). Many sequences previously assigned to HV0* are in fact best included in the HV0-195 clade. Possibly, further genotyping on larger samples may reveal new lineages within this branch. HV0e includes five individuals from our Italian dataset (i.e. three from Northern Italy and two from Southern Italy). Other individuals fall in separate lineages out of the 195 clade, including three Italian subjects from our dataset and other three for which we do not have geographic assignation (S1 Fig. and Fig. 5); two Italian individuals from Northern Italy share mutation 12425 in a branch here named HV0g.

**HV1** is characterized by the stable transversion A8014t. In the network, it shows traces of a past expansion without central reticulations and a distinct small starlike pattern, suggesting a further recent expansion within the HV1b branch that involves Eastern European individuals (S8 Fig.). A lineage called HV1a'b'c' by automatic haplogroup assignment and characterized by a mutation in the coding region at position 3906 (as well as by the unstable 16129), is present in three Italian individuals, two of them from Northern Italy (i.e. from Aviano and Vicenza); this branch, which coalesces at around 11 kya according to the BEAST tree, is here named HV1e. Finally, one individual from Sicily which belongs to HV1b is characterized by 10 exclusive mutations.

**HV2** is clearly monophyletic and it is characterized by four mutations in the coding region, namely T7193C, A9336G, T11935C, and C12061T. It includes the subclade HV2a, characterized by the coding mutation G5147A. Both branches are clearly separated from the core of the HV variation, as pointed out also by the network (S10 and S11 Figs.). This branch is represented by sequences from Eastern Europe, Caucasus, North/Western Europe and Italy (both Northern and Southern regions), so that we cannot pinpoint a specific candidate geographic area for its origin and spread. Nevertheless, an apparent early branching includes three Italian individuals from Como and Enna, as well as one from India. Close to HV2, characterized by the A73G! mutation, we also find some isolated sister branches that were previously defined by the automatic assigner as HV9c. These share the recurrent mutation G73A (characteristic also of R0). These samples do not harbor the mutation G8994A characteristic of HV9 and appear to be HV-73 individuals with private mutations. In the network, two of these individuals are separated by a reticulated core, so that their assignation remains problematic, as mentioned in the text (S10 Fig.). Nevertheless, in the network with recurrent positions downweighted, these two are confirmed to be near HV2, in proximity to the root (S11 Fig.).

**HV12** is another monophyletic clade, characterized by the G13889A mutation. It is subdivided into the branches HV12a and HV12b, the latter being the only one that is characterized by a coding mutation (i.e. A15682G). The BEAST tree in S2 Fig. with full sequence data cannot resolve the clade as monophyletic and separate the two branches in the HV core, distinguishing HV12a first. This problem persists also in the Network (S12 and S13 Figs.).

**HV5** appears as a monophyletic clade in our trees and networks. It was previously defined by the mutations C12133T and A13105G! and includes individuals from Belorussia, Latvia and Poland. It coalesces around 11 kya according to S3 Fig.

Three individuals sharing coding mutation 9039 could represent a new branch here named HV18. Two of these individuals come from Italy and one comes from the Caucasus.

The block characterized by the mutation **16311** appears to be consistent in our reconstructed phylogenies (S2 and S13 Figs.), except when recurrent mutations are downweighted (S13 Fig.). The network with positions downweighted better resolves reticulation at the root and the unity of the other less represented haplogroups HV6, HV7, HV8, HV9, HV10, HV11, HV14, HV15, HV16 and HV17. We consistently find the T16311C! mutation in haplogroups HV6, HV7, HV8, HV9, HV10, HV11, HV14, HV15, HV16 and HV17, as described by literature, with the exception of individuals assigned to HV9c, in which it is expect to back-mutate according to Phylotree. In our dataset, the lack of G8994A suggests a new denomination for these individuals, as discussed before.

References

1. Gómez-Carballa A, Olivieri A, Behar DM, Achilli A, Torroni A, Salas A. Genetic continuity in the franco-cantabrian region: New clues from autochthonous mitogenomes. PLoS One. 2012; 7:e32851.
2. Soares P, Ermini L, Thomson N, Mormina M, Rito T, Röhl A, et al. Correcting for purifying selection: an improved human mitochondrial molecular clock. Am J Hum Genet. 2009; 84:740–759.
