# Supplementary material for: Fine Dissection of Human Mitochondrial DNA Haplogroup HV Lineages Reveals Paleolithic Signatures from European Glacial Refugia
Source: PLoS One. 2015 Dec 7;10(12):e0144391. doi: 10.1371/journal.pone.0144391 (PMC4671665; doi:10.1371/journal.pone.0144391)

S3 Fig. Phylogenetic tree with all the 316 sequences of the dataset.
Realized in BEAST, after imposing monophyly of major haplogroups branches HV0, HV1, HV4,

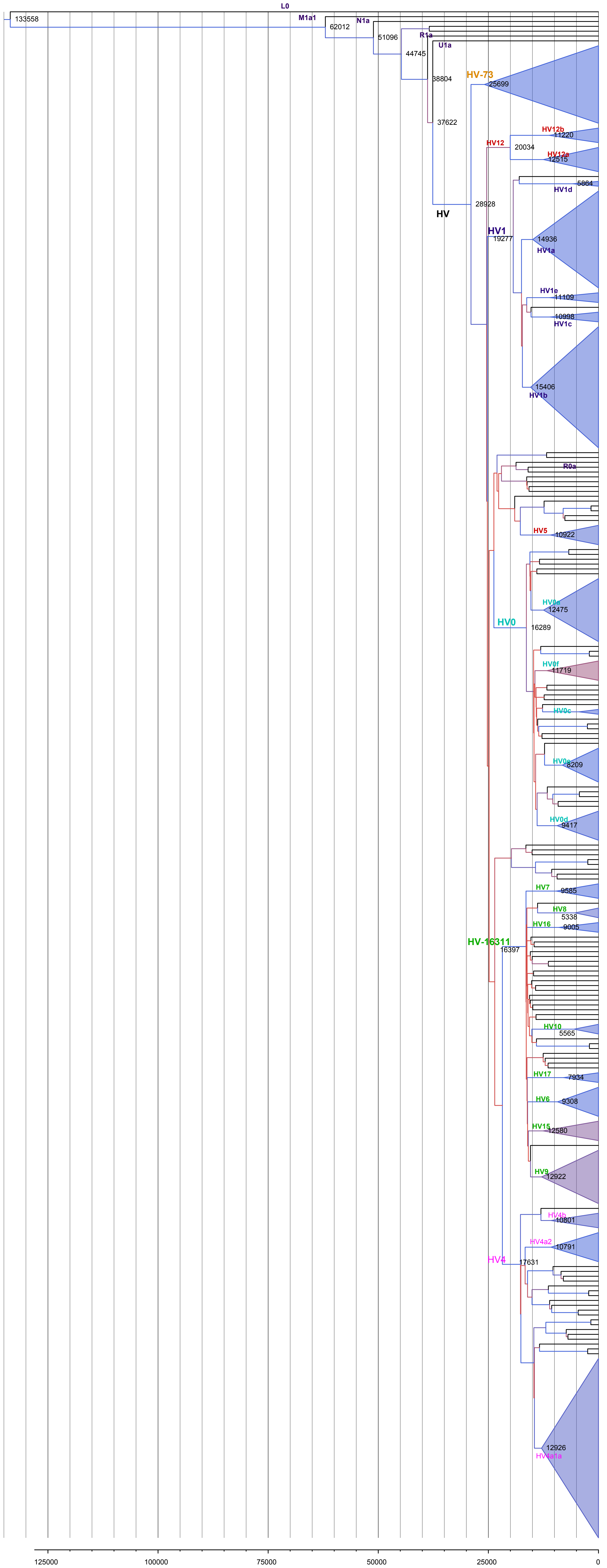

Supplement: S3 Fig — Realized in BEAST, after imposing monophyly of major haplogroups branches HV0, HV1, HV4, HV-73 and HV-16311. (PDF) [file pone.0144391.s003.pdf]
